# Spatial accessibility of primary health care utilising the two step floating catchment area method: an assessment of recent improvements

**DOI:** 10.1186/1476-072X-11-50

**Published:** 2012-11-16

**Authors:** Matthew R McGrail

**Affiliations:** 1 Gippsland Medical School, Monash University, Northways Road, Churchill, VIC, 3842, Australia; 2 Centre of Research Excellence in Rural and Remote Primary Health Care, Gippsland, Australia

**Keywords:** Spatial accessibility, Primary health care, Rural health, Access to health care, Service catchments, Medical geography

## Abstract

**Background:**

The two step floating catchment area (2SFCA) method has emerged in the last decade as a key measure of spatial accessibility, particularly in its application to primary health care access. Many recent ‘improvements’ to the original 2SFCA method have been developed, which generally either account for distance-decay within a catchment or enable the usage of variable catchment sizes. This paper evaluates the effectiveness of various proposed methods within these two improvement groups. Moreover, its assessment focuses on how well these improvements operate within and between rural and metropolitan populations over large geographical regions.

**Results:**

Demonstrating these improvements to the whole state of Victoria, Australia, this paper presents the first comparison between continuous and zonal (step) decay functions and specifically their effect within both rural and metropolitan populations. Especially in metropolitan populations, the application of either type of distance-decay function is shown to be problematic by itself. Its inclusion necessitates the addition of a variable catchment size function which can enable the 2SFCA method to dynamically define more appropriate catchments which align with actual health service supply and utilisation.

**Conclusion:**

This study assesses recent ‘improvements’ to the 2SFCA when applied over large geographic regions of both large and small populations. Its findings demonstrate the necessary combination of both a distance-decay function and variable catchment size function in order for the 2SFCA to appropriately measure healthcare access across all geographical regions.

## Introduction

Access to health care is widely accepted internationally as a key goal in meeting the health needs of individuals [1-4]. However, assessing the extent to which adequate access to health care services is achieved is difficult because there is no single agreed definition of access [5-8]. Healthcare access is such a complex concept that Norris and Aiken [9] went as far as to state that *“It is as if everyone is writing about ‘it’ [access] but no one is saying what ‘it’ is”.*

A fundamental problem of defining access is its status as both a noun and a verb [10], thus healthcare access can refer both to the potential for use as well as the act of using healthcare. Furthermore, access is multidimensional with specific access barriers covering a range of spatial and aspatial dimensions [11-13], making it difficult to operationalise. Health service planners have tended to adopt Penchansky and Thomas’ [13] five main dimensions of access – specifically availability, accessibility, affordability, accommodation and acceptability. As a result, healthcare access indicators vary immensely, and may be capturing but not limited to the availability of care, the ability to get to and pay for available care, or the act of seeking and utilising available care. One common approach to evaluating access to health care is through measuring spatial accessibility [10,14-16]. Spatial accessibility provides a summary measure of two important and related components of access - firstly the volume of services provided relative to the population’s size and secondly the proximity of services provided relative to the location of the population. This paper focuses on one such measure of spatial accessibility, the two-step floating catchment area (2SFCA) method and evaluates its many recent ‘improvements’.

## Background

### Spatial accessibility and the two-step floating catchment area (2SFCA) method

The accurate measurement of spatial accessibility to health care is problematic chiefly because there is seldom any predetermined assignment or single pathway between individuals and specific health care services. That is, in most western societies individuals are free to access health care wherever and from whomever they choose. Thus an assessment of available services relative to the needs of the population specific to a local area is challenging. This is especially true for primary health care services, the key facilitator of access within most international health care systems [17,18], which co-exist in a network of overlapping catchments ‘competing’ for the population’s utilisation of their services.

The two-step floating catchment area (2SFCA) method, pioneered by Luo and Wang [11,15,19], emerged from a background in which the shortcomings of existing measures of spatial accessibility were readily apparent. In particular, fundamental weaknesses of provider- or physician-population ratios (PPRs) are well-recognised [10,14,15], which fail to include both cross-border movement between boundaries and distance decay within boundaries, but most significant is their restriction to using fixed geographical or administrative boundaries such as counties or postcodes. The 2SFCA method builds upon the framework of PPRs, but instead uses floating catchment areas which overlap, thereby enabling the modelling and measurement of ‘real-life’ healthcare access behaviour with unrestricted utilisation. The size of the catchment is determined by a choice of maximum travel time (or distance), where all services (or populations) within that catchment are considered accessible and equally proximate to that particular population (or service), whilst all locations outside of the catchment are not accessible.

The process for calculating the 2SFCA method is relatively straightforward. Step 1 of the 2SFCA method determines what populations (k) of size P_k_ are located within the catchment of each service provider (j) of volume S_j_, thus defining the provider-to-population ratio R_j_ within a service catchment (that is, the potential service demand). Step 2 then ‘allocates’ these service ratios to the population by determining which services (j) are located within the catchment of each population (i), and aggregating the Step 1 (R_j_) scores to calculate a location’s access (A_i_). The only decision required in applying the 2SFCA method is the catchment size (d_max_), which is then applied at both Steps 1 and 2. This method has been utilised in this form or with minor modifications only, within the last six years by many different studies [20-31].

Step 1: For each service (j), R_j_ = S_j_/∑_k∈ {djk <dmax}_P_k_

Step 2: For each population (i), A_i_ = ∑ _j∈ {dij <dmax}_R_j_

The following assumptions are made regarding application of the 2SFCA method:

Service providers are represented by their geocoded organisational address (latitude, longitude). Aggregating service counts to some administrative boundary (e.g. town, county, postcode) will simplify its computation, but can greatly reduce its sensitivity to small-area discrimination.

Population (aggregated) groups are represented through a single location (centroid, usually geometric or population-weighted), based on some larger administrative boundary. Usage of smaller areal units enables more accurate small-area measurement of ‘local’ access, but also greatly increases computation complexity.

Population-provider proximity (d) is measured as time or distance separation (point-to-point) through some transport network (roads, public transport). Euclidean distance can also be used to approximate proximity; however, this results in a moderate loss of accuracy.

Whilst the greatest strength of the 2SFCA method is it overcomes the restriction of using only pre-defined regional boundaries, this improvement alone does not address two major weaknesses still apparent in its framework. Firstly, distance-decay is assumed to be negligible within a catchment, something which is clearly not the case in large geographical regions where populations are widely dispersed, and catchments therefore are quite extensive. Secondly, catchments are assumed to be the same size for all populations *and* for all services.

Over the past five years, several authors have developed methodological ‘improvements’ to address these weaknesses characterising the 2SFCA method. This paper assesses these improvements to the 2SFCA method by evaluating their effectiveness when applied to primary health care access. Moreover, this assessment will focus on how well these improvements operate within and between rural, regional and metropolitan populations over large geographical regions. Health policies are mostly applied at national or state levels which require methodologies to work across diverse and large geographies. However, to date most 2SFCA method improvements have been demonstrated only within small or localised areas.

### Improvement 1 – addition of distance decay function

Without the addition of a distance decay function, there is widespread agreement that the 2SFCA method is deficient [32-35]. Its omission is equivalent to accepting that distance (or time) is a negligible barrier within a catchment, an unlikely scenario for geographically large countries and given our use of a maximum catchment of 60 minutes in this paper. Within a *service* catchment (Step 1), distance-decay omission means that any service is equally likely to be delivering services to both populations very close by and those up to the catchment boundary (60 minutes). Within a *population* catchment (Step 2), distance-decay omission means that individuals are equally likely to be accessing services from both nearby and up to the catchment boundary (60 minutes). Whilst omission of both of these may be acceptable for a small scale model in a densely populated area (such as within major cities), it is clear that a distance-decay function is crucial in sparsely populated (rural) areas where problems associated with poor access to health care services are known to be a major factor contributing to the poorer health status of population in these areas [36,37].

Currently there is little empirical evidence to guide the choice of one decay function over another. Wang [38] defined six different distance-decay functions, where the crude 2SFCA method is defined by its use of a binary discrete function with no decay within a catchment and complete decay outside of a catchment. Luo & Qi [34] developed what they called the ‘enhanced’ 2-step floating catchment area (E2SFCA) method, where catchments are broken into 3 discrete zones (0–10 minutes; 11–20 minutes; 21–30 minutes) with constant weightings (w<=1) applied to the accessibility within each zone. Subsequently, some authors have accepted the E2SFCA method as the new ‘standard’ 2SFCA method [21,32,39], whilst Wan et. al. [40] extended this approach by adding a 30–60 minutes zone. Drawing on Wan and the Gaussian distribution [40,41], two sets of weightings are tested in this paper for these four time barrier zones, relating to either fast or slow decay:

Fast step-decay: weightings (w) = 1, 0.60, 0.25, 0.05

Slow step-decay: weightings (w) = 1, 0.80, 0.55, 0.15.

In applying this zonal or step approach to large geographical areas, the key criticism remains - specifically that accessibility weightings are equal within each zone and there is a sudden step (drop) at the edge of each zone [32,33], something which does not match real utilisation behaviour. Resultantly, many authors have developed distance-decay functions which are smoother and continuous in their decay [32,35,42,43]; however without any empirical evidence, it is unclear which function is the most appropriate to use [38]. This paper tests one such continuous weighting function:

Continuous decay: weightings (w) = 1 for the first 10 minutes, w = 0 for more than 60 minutes, and w = ((60-d)/(60–10))^1.5 for distance/time (d) between 10 and 60 minutes.

**Figure 1:**
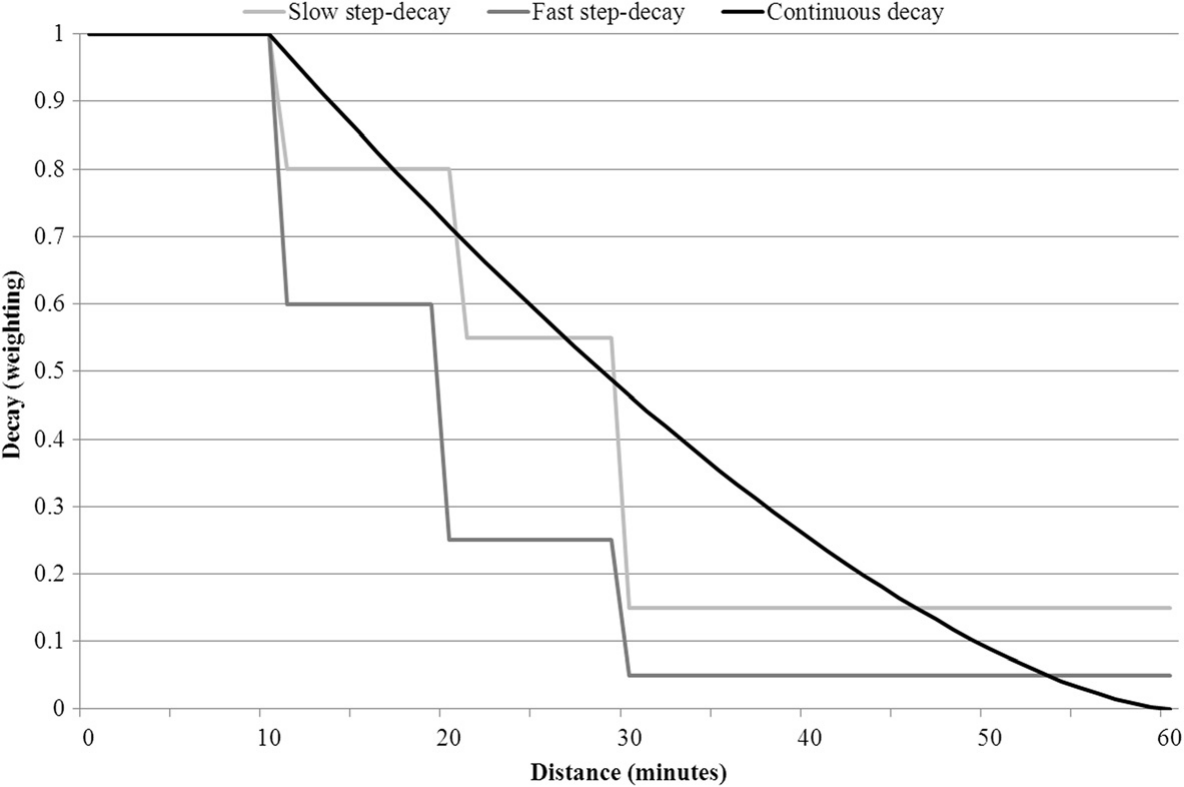
Decay function (weighting) versus distance (minutes).

Previous testing suggested 1.5 was an appropriate weighting factor [35], though higher values such as 2 would achieve a quicker decay rate. Figure 1 shows the relative weightings of the three decay functions to be tested.

### Improvement 2 – addition of variable catchment size function / variable inclusion of distance decay

One feature of applications of the 2SFCA method by different authors is their choice of different catchment sizes, with those in metropolitan settings generally using significantly smaller catchment sizes than for rural settings. Metropolitan and rural regions have very different settlement patterns, meaning that the appropriate catchment size for different regions is likely to vary greatly. Despite this, to date only two research groups have investigated the use of a variable catchment size function or the related variable inclusion of a distance decay function [35,44], with each using very different methods for defining their population and service catchment sizes.

Variable *population* catchment size (Step 2) is conceptually simple – individuals can travel further (or have a higher likelihood of travelling further) to access healthcare if nearby services do not meet their needs [45]. In metropolitan areas, services and populations are densely located so that, typically, most individuals will access services in close proximity because their requirements are met. In contrast, rural services are more dispersed, so that rural populations commonly access services beyond their immediate community. McGrail and Humphreys [46] modelled this rural/metropolitan distinction by limiting population catchment sizes to those containing the nearest 100 services (up to a maximum of 60 minutes, with a minimum catchment of 10 minutes). Similarly, Luo and Whippo [44] defined a minimum population catchment size of 10 minutes; however their approach was very different as a result of incrementally increasing the population catchment size (up to a maximum of 60 minutes) until a minimum provider-to-population ratio (1:3500) is reached.

In metropolitan settings, both of these approaches ensure that the population catchment size is close to the minimum level of 10 minutes. In rural settings, McGrail’s approach will have negligible effect because most rural populations will not have 100 services within a catchment of up to 60 minutes; however in metropolitan-fringe settings the effect of this approach will be most noticeable because the capping of 100 nearest services will vary greatly. Luo’s approach does not distinguish between geographical settings; rather its effect is dependent on the local access level with populations modelled as only travelling further if their local access is below a minimum level.

Variable *service* catchment size (Step 1) is conceptually more difficult, with three broad scenarios defining different catchment areas requirements: (1) Metropolitan services provide access mostly to only their local neighbourhood; (2) Services in metropolitan-fringe areas or larger rural communities will frequently serve populations located well beyond the local community; (3) In contrast, services in small rural communities are generally not providing access for populations in larger nearby communities, who have adequate access within their immediate community.

McGrail and Humphreys proposed a method for variably applying distance decay at Step 1 across all regions, based on differentiating local population distributions [35]. Their four sequential rules (Table 1) are broadly designed to follow the three scenarios described in the previous paragraph and enable the 2SFCA method to dynamically determine where distance-decay should be applied (and thus reduce the size of the population being served).

**Table 1:** McGrail and Humphrey’s rules to define the variable application of distance-decay to service catchments (Step 1)

**Rule**	**Explanation**	**Outcome**
1. Population within 10 minutes of the service	Initial (local neighbourhood) catchment without distance decay	No decay
2. Population linked to their nearest 25 services	Services likely to provide access to populations (beyond 10 minutes) that have few alternative options	No decay
3. Population <5000 *and* < 0.5 population of the Service town	Services in larger towns likely to provide access to significantly smaller nearby populations, but not vice-versa	No decay
4. *All other scenarios*	Services less likely to provide access to populations as distance increases.	Decay

Luo and Whippo [44] took a much simpler approach to defining service catchments by increasing its size incrementally (starting at 10 minutes) until the catchment population reaches 500,000 (note: for this paper, 250,000 or 250K was considered a more appropriate size, and tested henceforth). It is clear that Luo’s approach will have minimal or no effect on service catchments in sparsely populated (rural) areas, where the catchment population will be significantly less than 250K.

## Methods

### Study area and data requirements

**Figure 2:**
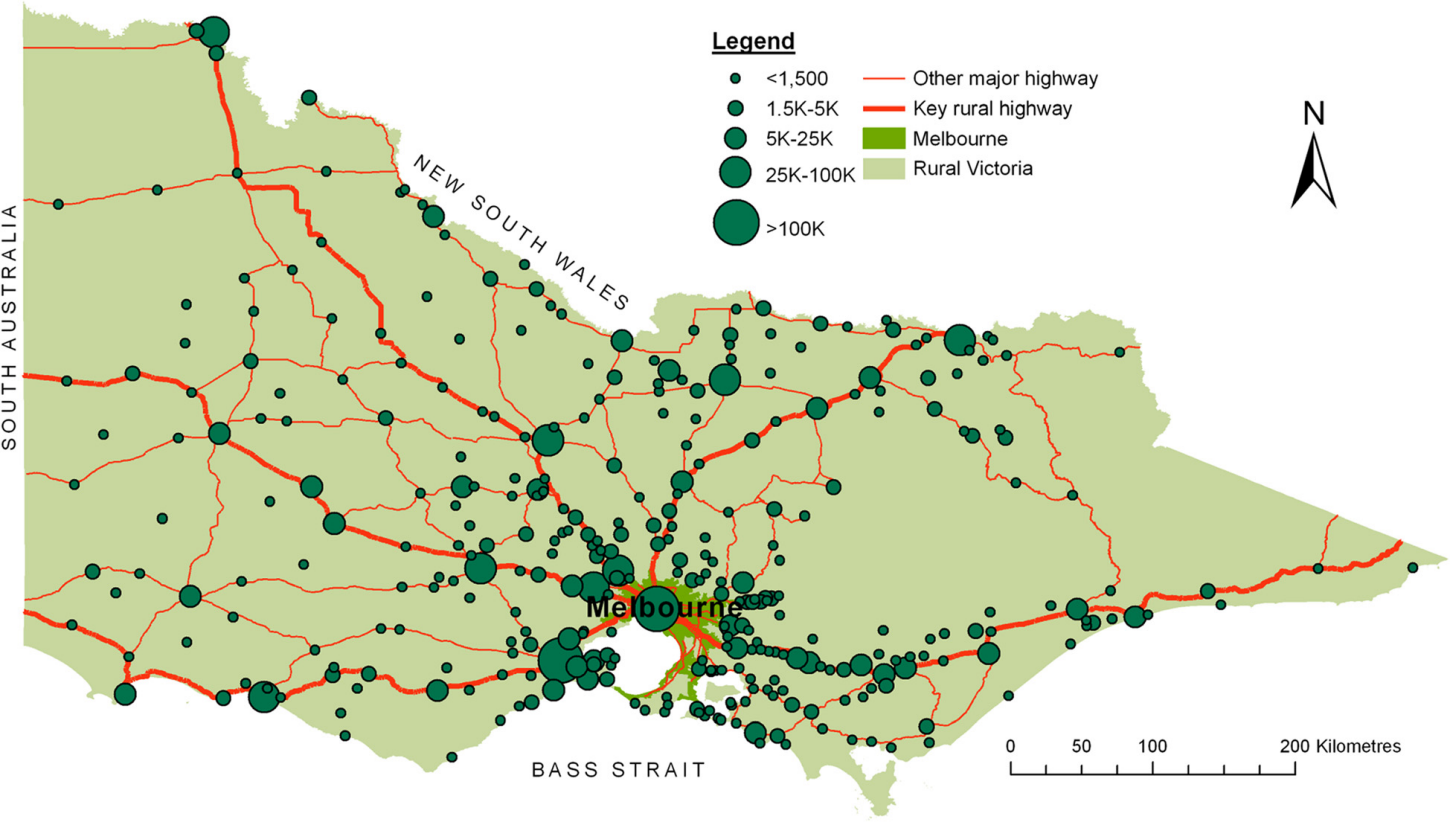
Distribution of population centres within Victoria.

To evaluate the 2SFCA method improvements, access scores are calculated using general practitioner (GP) service data in the state of Victoria, Australia (see Figure 2). Victoria has a total area of 227,000 square kilometres and 2011 population in excess of 5½ million (of which some 35% reside outside of its major city of Melbourne). Firstly, population size and location data were obtained from the 2006 national census, using the smallest geographical unit of ‘collection districts’ (CDs) which contain an average population of approximately 500 residents. Secondly, lists of GP locations (street address) and full-time equivalence counts were obtained from the *Medical Directory of Australia* (2006 records), a dataset which is updated every six months and promoted as being over 99% accurate and over 90% complete. Thirdly, proximity between geo-coded GP locations and population (CD) centroids was calculated using road networks and the ‘Closest Facility’ tool of the Network Analysis module of ArcView 9.1, with travel time impedance captured by combining road section lengths and approximate section travel speeds. Based on the notion of the ‘golden hour’ rule [47,48], a maximum catchment size of 60 minutes was used, and bordering data up to one catchment in width, from the neighbouring states of New South Wales and South Australia, were also included to take account of cross-border access at the edges of the study area.

**Figure 3:**
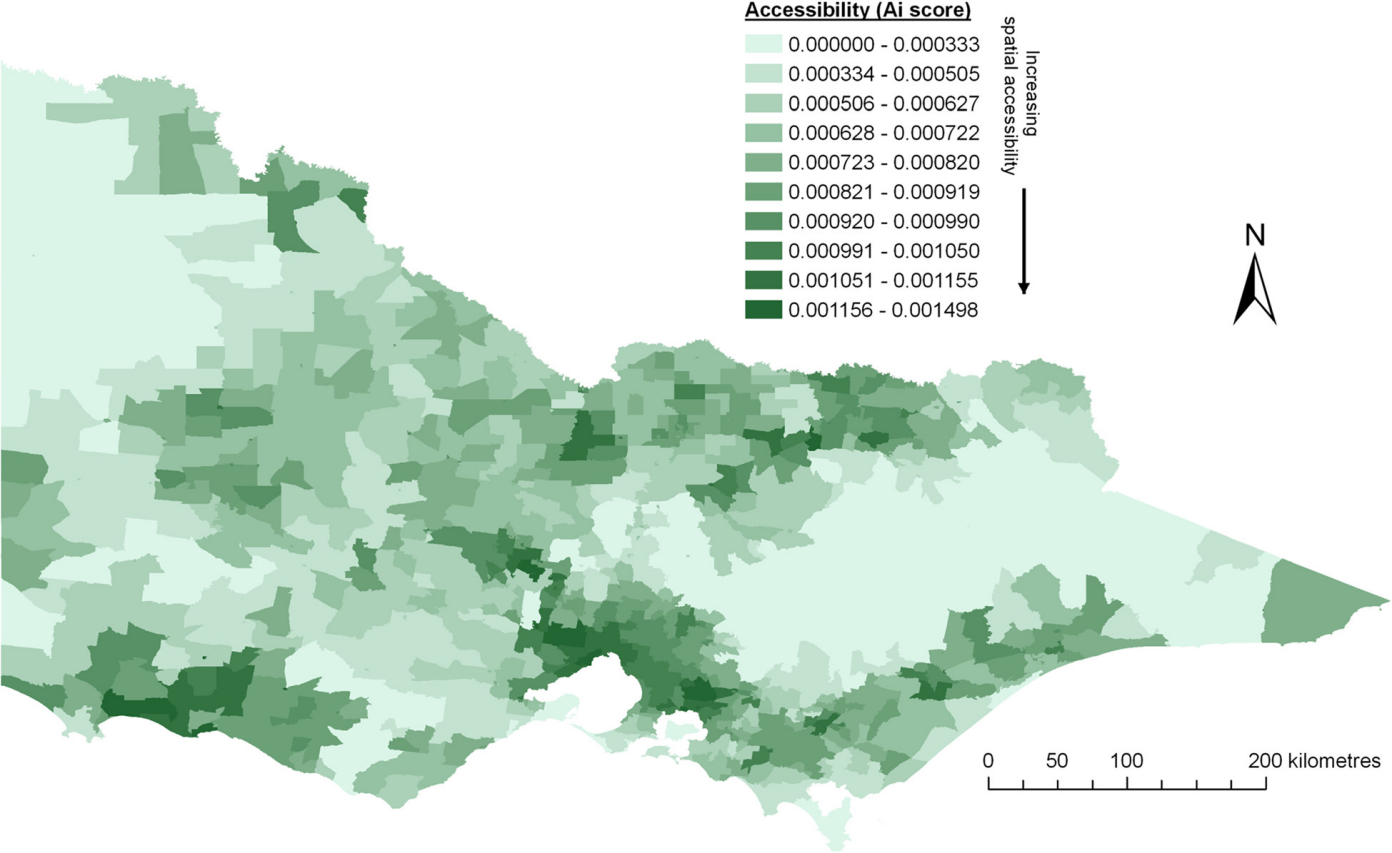
Results from applying the crude 2SFCA method across all of Victoria.

Resultant access scores were calculated using the original (crude) 2SFCA method in combination with tested improvements as previously described. As a baseline comparison, Figure 3 shows the results of applying Luo and Wang’s crude 2SFCA method across the whole state of Victoria, that is, before the inclusion of any ‘improvements’. All access scores are assessed against a five-level community population size scale, which align closely with natural break points of primary care service provision in Australia [49,50]. Specifically, these are: (1) very small rural: <1,500 residents (none or limited GP services within community); (2) small rural: 1,500–4,999 residents (narrow choice of GP services within community); (3) medium rural: 5,000–24,999 residents (moderate choice of GP services within community); (4) large rural: 25,000–99,999 residents (wide choice of GP services within community); (5) metropolitan: >100,000 residents.

## Results

### Outcomes: improvement 1 – addition of distance decay function

**Figure 4:**
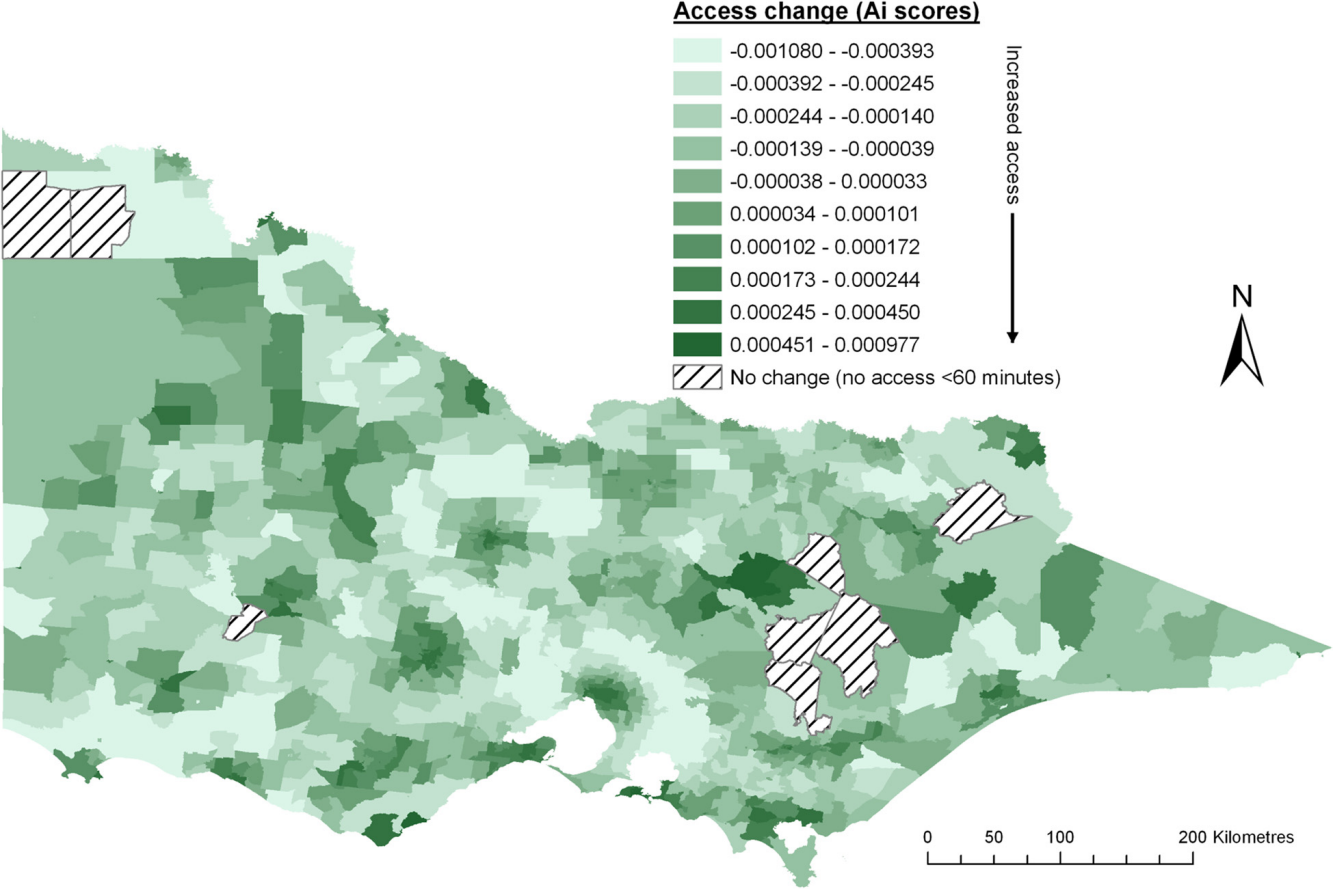
Change after addition of slow step distance decay function (compared to crude 2SFCA method).

Table 2 and Figure 4 show the result of integrating each of three different distance-decay functions within the 2SFCA method at both Step 1 and Step 2, which are summarised across five population size groupings. Within the four rural groupings (<100K), there is a consistent pattern between decreasing population size and increasing proportion of lower access scores (see Table 2) following the addition of any distance-decay function. This is most apparent in the very small rural group (<1.5K), where most populations do not have a local resident doctor and thus are most affected by the introduction of a distance-decay function. As a result, a large shift of access scores towards the lowest 4 categories (7^th^ – 10^th^) was seen in <1.5K communities for all three distance-decay functions. In contrast, the large rural group (25-100K) saw a shift of access scores towards the higher access categories (2^nd^ – 4^th^) and a decrease in the poorer access (5^th^ – 7^th^) categories. The addition of the distance decay function has strengthened the association between both large populations and increased access, and between small populations and decreased access.

**Table 2:** Distribution of crude access (2SFCA) scores and resulting change of access scores with the addition of three different distance-decay functions, by population size

	**Access category (A**_**i**_**score range)**									
	**1>0.0012**	**2>0.001**	**3>0.0009**	**4>0.0008**	**5>0.0007**	**6>0.0006**	**7>0.0005**	**8>0.0004**	**9>0.0003**	**10<0.0003**
**Crude access (2SFCA) score distribution ^1**										
**>100K**	17	2012	1049	17	64	59	10	23	0	10
**25-100K**	28	0	34	61	38	99	49	0	0	0
**5-25K**	12	44	66	55	30	27	23	26	3	18
**1.5-5K**	0	32	28	34	24	15	19	7	3	11
**<1.5K**	10	44	67	92	66	85	61	44	34	30
**Slow step-decay (net change from crude 2SFCA) ^2**										
**>100K**	992	−1115	−484	182	302	4	42	46	29	1
**25-100K**	−28	63	45	21	−32	−45	−49	25	0	0
**5-25K**	−2	4	−16	−10	−12	19	44	−8	−2	−18
**1.5-5K**	6	−17	−11	−11	3	7	−6	15	20	−5
**<1.5K**	−7	−26	−36	−45	−2	−21	27	38	28	44
**Continuous decay (net change from crude access scores) ^2**										
**>100K**	694	−878	−374	306	190	−23	11	36	37	1
**25-100K**	−28	51	45	33	−32	−45	−49	25	0	0
**5-25K**	−3	17	−19	2	7	28	7	−25	5	−18
**1.5-5K**	4	−18	−11	−2	3	8	−2	18	1	−2
**<1.5K**	−6	−21	−38	−35	12	3	6	30	13	37
**Fast step-decay (net change from crude access scores) ^2**										
**>100K**	1039	−1338	−344	165	142	188	84	49	14	1
**25-100K**	−25	113	54	−44	−2	−99	−21	0	25	0
**5-25K**	3	32	−19	−17	−20	46	−3	−20	15	−17
**1.5-5K**	10	−12	−9	−12	−2	−2	−9	22	10	6
**<1.5K**	−1	−23	−45	−59	−16	−25	−10	44	39	95

All figures within the table are ‘000s.

^1: These values represent the size (‘000s) of the population with these access scores. Row totals correspond to the total Victorian population residing in the 5 population size groups.

^2: These values represent the net population change (‘000s) within each population size group to the corresponding access scores following the addition of each distance-decay function. Negative values indicate a net drop in the number of residents with access scores in that category. All row totals equal 0.

The three distance-decay functions revealed only minor differences to the change of access scores in Table 2 across the four rural groupings, though it must be noted these are net population changes and don’t necessarily apply to the same geographic regions. In particular, there is a high correlation for changes between the slow step-decay and continuous decay functions. Figure 1 shows that these functions have similar shapes thus their level of similarity in resultant access scores is not altogether surprising. Closer assessment reveals a slight bias towards higher access scores in rural populations using the continuous function, which is likely due to its slower decay beyond 30 minutes. Specific to the fast step-decay function, two moderate differences are observed. Firstly, in the smallest rural group (<1.5K) there is a considerable increase in the size of the poorest access group. This occurs because as distance-decay is applied more quickly (at Step 2), populations who are required to travel further to access distant services are affected most and thus their access scores decrease most. The second noticeable difference in rural populations is a small increase in the two highest access categories (1^st^ and 2^nd^). This occurs because a faster distance-decay (applied at Step 1) causes decreased demand from distant populations (that is, it decreases the denominator in Equation 1), the result of which is inflated access scores.

The introduction of a distance decay function has had the greatest effect (population size) within metropolitan centres (>100K). This is largely explained by the crude 2SFCA method allocating most metropolitan populations to only the 2^nd^ and 3^rd^ access categories. After applying any one of the distance-decay functions, the highest access category has increased greatly (694K – 1039K), mostly at the expense of the 2^nd^ access category whilst the 4^th^ and 5^th^ categories have increased significantly (142K – 306K) at the expense of the 3^rd^ access category. Meanwhile, the lowest 5 access categories for metropolitan populations have all increased slightly, with the fast step-decay function seeing the largest increase in poorer access scores. Notably, Figure 4 demonstrates a very strong geographical pattern within metropolitan Melbourne, with the largest access gains located in the geographical centre whilst the largest losses are all located in the outer-urban / rural-fringe areas with a clear concentric pattern of decreasing ‘access’ from the centroid to the metropolitan-fringe. This suggests that with the addition of distance-decay function, the 2SFCA method is now largely measuring ‘choice’ in metropolitan areas. In the next section, which assesses the addition of a variable catchment size function, the slow step-decay function has been included.

### Outcomes: improvement 2 – addition of variable catchment size function / variable inclusion of distance decay

**Figure 5:**
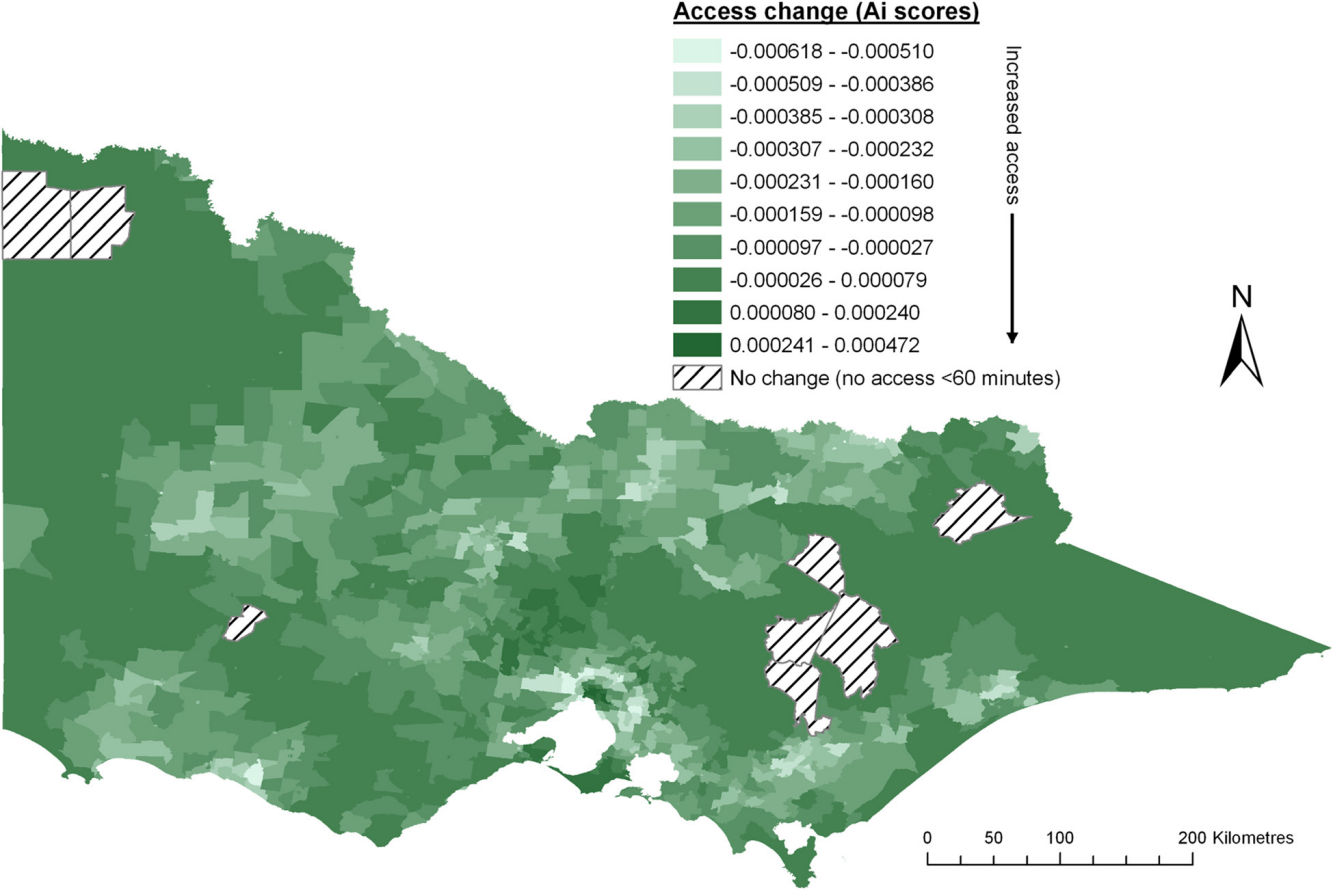
Change after addition of Luo and Whippo’s variable catchment size function (compared to crude 2SFCA method with slow step-decay).

**Figure 6:**
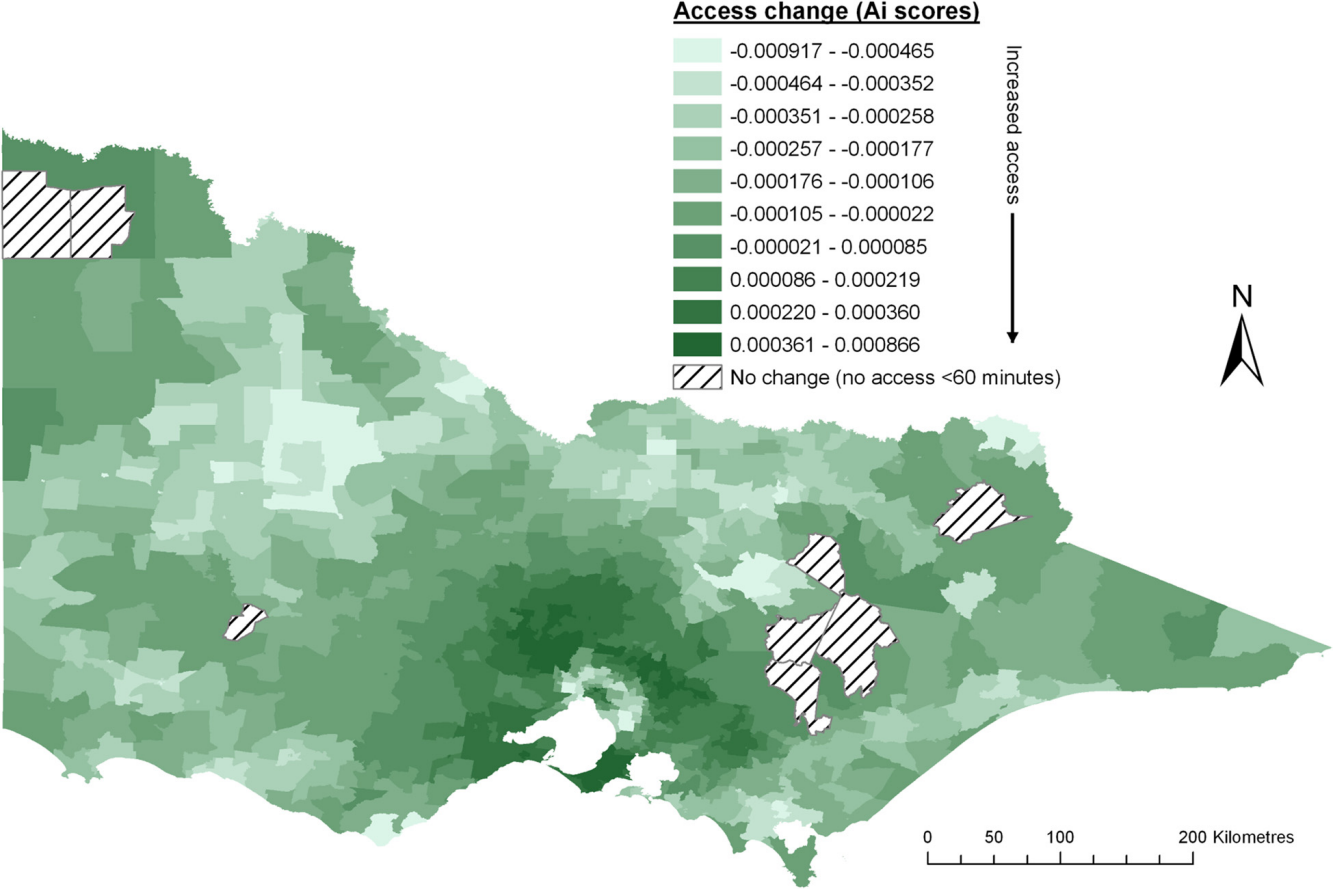
Change after addition of McGrail and Humphreys’ variable catchment size function (compared to crude 2SFCA method with slow step-decay).

Figures 5 and 6 respectively show the change to access scores across different geographic regions with the addition of either Luo’s or McGrail’s variable catchment size function. Outcomes from applying these functions are further summarised across five population size groupings in Table 3 and many differences between these functions are apparent.

**Table 3:** Resultant change of access scores with the addition of two different variable catchment size functions, by population size

	**Access category (A**_**i**_**score range)**									
	**1>0.0012**	**2>0.001**	**3>0.0009**	**4>0.0008**	**5>0.0007**	**6>0.0006**	**7>0.0005**	**8>0.0004**	**9>0.0003**	**10<0.0003**
**Luo and Whippo’s approach (net change from crude access scores with slow-step decay)^1**										
**>100K**	−250	−600	−521	127	−74	255	624	326	125	−11
**25-100K**	0	−63	−79	−55	67	67	34	29	0	0
**5-25K**	−10	−47	−44	−25	21	46	13	17	28	1
**1.5-5K**	−6	−9	−6	−11	−13	5	32	0	1	7
**<1.5K**	−3	−15	−29	−30	−38	1	18	34	32	29
**McGrail and Humphreys’ approach (net change from crude access scores with slow-step decay)^1**										
**>100K**	−406	−480	−313	201	363	452	185	37	−29	−11
**25-100K**	0	−35	−47	39	31	8	29	−25	0	0
**5-25K**	−10	−41	−18	9	41	6	−16	14	10	4
**1.5-5K**	−6	−7	−15	7	−6	10	11	13	−8	0
**<1.5K**	−2	−11	−17	−14	7	4	−13	2	13	30

All figures within the table are ‘000s.

^1: The base (comparison) model is a crude 2SFCA method with the addition of a slow step-decay function. These values represent the net population change (‘000s) within each population size group to the corresponding access scores following the addition of each distance-decay function. Negative values indicate a net drop in the number of residents with access scores in that category. All row totals equal 0.

Arguably the greatest need for a variable catchment size function is within metropolitan-fringe areas. It was seen in Figure 4 that the resultant change from applying a distance-decay function alone in these areas was consistently decreasing access scores moving out from the city centre to metropolitan-fringe populations. This is chiefly because the 2SFCA method, prior to adding a variable catchment size function, models metropolitan-fringe locations as being ‘swamped’ by inner-metropolitan populations travelling out to these fringe (and nearby rural) locations, guaranteeing their lower access scores. Both functions (Luo’s and McGrail’s) rectify this problem to varying degrees. McGrail’s Step 1 distance-decay function rules (Table 1) have reversed much of the drop in access scores in metropolitan-fringe areas, though its validity is difficult to assess. Luo’s simpler approach too increases access in many metropolitan-fringe areas, but is less effective for two reasons. Firstly, their constant use of a 250K service catchment ensures metropolitan-fringe populations continue to ‘swamp’ nearby rural services. Secondly, their function which expands the population catchment only until a minimum access level (<1:3500) is reached is problematic because in nearby rural areas it is highly likely that local populations can and will choose to travel a little further into metropolitan-fringe areas to access services. Thus the ‘real’ access score should be larger in these areas than their function allows.

In all rural population groups, Table 3 shows that Luo’s approach has reduced access levels for more of the population compared to McGrail’s approach. For example, in 5-25K rural towns Luo’s model has increased a net total of 126K into the lowest 6 access categories (<0.0008) whilst the comparative net movement in McGrail’s model to the same 6 access categories is 59K. This pattern is consistent in all five population size groupings, to various degrees and is predominantly due to Luo’s use of the population catchment size minimum PPR rule (>1:3500 as applied to Step 2), whilst their Step 1 rule has virtually no effect in rural areas. Interestingly, the opposite application occurs in McGrail’s model with their rule of limiting the population catchment to the nearest 100 services (as applied to Step 2) having virtually no effect in rural areas, whilst their Step 1 rules (Table 1) greatly affect rural access scores.

Visually, changes in rural access scores are quite different using either Luo’s (Figure 5) or McGrail’s (Figure 6) approach. Closer investigation of Figure 6 (McGrail’s approach) reveals that many areas showing the largest decrease in access accord directly with areas of largest increase in access in Figure 4. That is, for many areas there is a direct ‘correction’ from first applying the distance-decay function, then adding the variable catchment size function. This is explained by the Step 1 rules (Table 1) which purposefully aim to improve the determination of when, or if, the distance-decay function should be applied. Resultant access changes from Luo’s approach (Figure 5) have no apparent relationship to the distance-decay function (Figure 4).

## Discussion

This paper has critically evaluated current and recent work by a number of different researchers who have developed new methodologies aimed at improving the 2SFCA method. Generally, these improvements aim to address one of two deficiencies of Wang and Luo’s original (crude) 2SFCA method: (1) accounting for distance decay within a catchment; and (2) enabling variable catchment sizes or variable application of distance-decay.

To date, most 2SFCA method improvements relate to the addition of a distance-decay function, with a general acceptance that its omission is highly problematic. This paper provides the first evidence of the effect of applying one of three related distance-decay functions, and most significantly their application across large geographic regions of both rural and metropolitan populations. Despite some criticisms of the step-decay function having a sudden drop in access at the edge of each zone, these results showed relatively minor differences when comparing the continuous and slow-zone functions, particularly in more sensitive rural areas. A continuous-decay function may intuitively be preferable to a step-decay function, but it is difficult to define an appropriately shaped function that matches ‘real’ behaviour of the population (chiefly because of poor empirical evidence for health care seeking behaviour). Figure 1 suggests that the decay weighting of the tested continuous function may be too slow, particularly for a distance barrier of 30–60 minutes. In contrast, the consequence of choosing a faster step-decay function in rural areas has (perhaps wrongly) increased the proportion of low-access scores within small rural areas (<25K) and somewhat increased the proportion of high access scores in larger rural areas (25-100K).

Importantly, the application of any distance-decay function without the simultaneous use of a variable catchment size function creates a strong concentric pattern of high to low access scores out through metropolitan-fringe and into nearby rural areas. This ‘overcorrection’ in metropolitan-fringe areas can be rectified by adding a variable catchment size function, so that more realistic movements of these populations are captured in the modelling. Furthermore, it is not clear that distance / time barriers are viewed consistently by all populations within different geographical settings, thus it is questionable whether the same distance-decay function would apply to all. For example, residents in isolated and vastly settled areas are likely to have a higher ‘tolerance’ of travelling further to access services versus residents in closely settled or largely populated areas.

The respective variable catchment size functions used by either McGrail and Humphreys or Luo and Whippo approach the problem from contrasting sides of access calculation. McGrail’s method limits population catchments (applied at Step 2) to the nearest 100 services (or minimum 10 minutes), whilst Luo’s method limits service catchments (applied at Step 1) to the nearest 250K residents (or minimum 10 minutes). Both of these approaches have full effect in metropolitan areas, no effect in rural areas and varying effect in metropolitan-fringe and nearby rural areas. Luo’s use of a minimum accessibility rule to define population catchment sizes (applied at Step 2) is problematic in how effectively it adjusts access scores. This was most apparent in metropolitan-fringe areas, where access scores were significantly lower using Luo’s method, but it is arguable whether these populations would readily travel a small distance further to access metropolitan-based services areas, and so their access scores have been capped inappropriately. McGrail’s approach to service catchments (applied at Step 1) does not use a single size for all populations, unlike Luo’s constant use of 250K, but instead defines a set of four rules which account for neighbouring populations and service competition. Whilst these rules attempt to better match ‘real’ supply behaviour, further independent assessment is required.

The key limitation to this study is the lack of available empirical data on ‘real’ health service access behaviour and its relationship to geography. The 2SFCA method requires assumptions of catchment size, distance-decay and the variable application of these across metropolitan and rural populations; however, to date most applications of the 2SFCA method have not been verified against empirical access behaviour data. An additional related limitation is that it’s not known whether this paper’s findings (based on the state of Victoria, Australia) readily translate to other geographical settings and countries.

Most academic papers describing methodological developments or improvements such as those for the 2SFCA method underestimate the importance of the specifics of the geography under consideration. One of the difficulties of calculating spatial accessibility is modelling across vastly different population densities and dispersions. A key strength of the 2SFCA method is that it can be readily applied to both metropolitan and rural populations. However, this ‘flexibility’ can also be one of its weaknesses when applied simultaneously across all geographies. Catchment size or distance-decay rules that readily apply in densely populated areas generally don’t apply in sparsely populated areas, and vice-versa. For this reason, the inclusion of a variable catchment size methodology, which is missing from the new standard E2SFCA method, is vital to the wider application of the 2SFCA method to health policies relating to healthcare access and primary health care service provision. This paper has provided further evidence relating to the inclusion and choice of both a distance-decay function as well as a variable catchment size function, highlighting in particular that large scale applications of the 2SFCA method require closer attention to their defining of population and service catchments.

## Competing interests

There are no competing interests.
